# Disruption of immune responses by type 1 diabetes exacerbates SARS-CoV-2 mediated lung injury

**DOI:** 10.1152/ajplung.00250.2024

**Published:** 2024-09-25

**Authors:** Sara Kass-Gergi, Gan Zhao, Joanna Wong, Aaron I. Weiner, Stephanie Adams Tzivelekidis, Maria E. Gentile, Meryl Mendoza, Nicolas P. Holcomb, Xinyuan Li, Madeline Singh, Yuru Huang, Alena Klochkova, Andrew E. Vaughan

**Affiliations:** ^1^Department of Biomedical Sciences, School of Veterinary Medicine, University of Pennsylvania, Philadelphia, Pennsylvania, United States; ^2^Institute for Regenerative Medicine, University of Pennsylvania, Philadelphia, Pennsylvania, United States; ^3^Penn Lung Biology Institute, University of Pennsylvania, Philadelphia, Pennsylvania, United States; ^4^Department of Medicine, Division of Pulmonary, Allergy and Critical Care, Perelman School of Medicine, University of Pennsylvania, Philadelphia, Pennsylvania, United States

**Keywords:** alveolar macrophages, COVID, lung injury, type 1 diabetes

## Abstract

COVID-19 commonly presents as pneumonia, with those most severely affected progressing to respiratory failure. Patient responses to SARS-CoV-2 infection are varied, with comorbidities acting as major contributors to varied outcomes. Focusing on one such major comorbidity, we assessed whether pharmacological induction of type 1 diabetes mellitus (T1DM) would increase the severity of lung injury in a murine model of COVID-19 pneumonia utilizing wild-type mice infected with mouse-adapted SARS-CoV-2. Hyperglycemic mice exhibited increased weight loss and reduced blood oxygen saturation in comparison with their euglycemic counterparts, suggesting that these animals indeed experienced more severe lung injury. Transcriptomic analysis revealed a significant impairment of the adaptive immune response in the lungs of diabetic mice compared with those of control. To expand the limited options available for tissue analysis due to biosafety restrictions, we also employed a new technique to digest highly fixed tissue into a single-cell suspension, originally designed for scRNA-Seq, which we then adapted for flow cytometric analysis. Flow immunophenotyping and scRNA-Seq confirmed impaired recruitment of T-cells into the lungs of T1DM animals. In addition, scRNA-Seq revealed a distinct, highly inflammatory macrophage profile in the diabetic cohort that correlates with the more severe infection these mice experienced clinically, allowing insight into a possible mechanism for this phenomenon. Recognizing the near certainty that respiratory viruses will continue to present significant public health concerns for the foreseeable future, our study provides key insights into how T1DM results in a much more severe infection and identifies possible targets to ameliorate comorbidity-associated severe disease.

**NEW & NOTEWORTHY** We define the exacerbating effects of type 1 diabetes mellitus (T1DM) on COVID-19 pneumonia severity in mice. Hyperglycemic mice experienced increased weight loss and reduced oxygen saturation. Transcriptomic analysis revealed impaired immune responses in diabetic mice, while flow cytometry and single-cell RNA sequencing confirmed reduced T-cell recruitment and an inflammatory macrophage profile. In addition, we introduced a novel technique for tissue analysis, enabling flow cytometric analysis on highly fixed tissue samples.

## Graphical abstract

**Figure:**
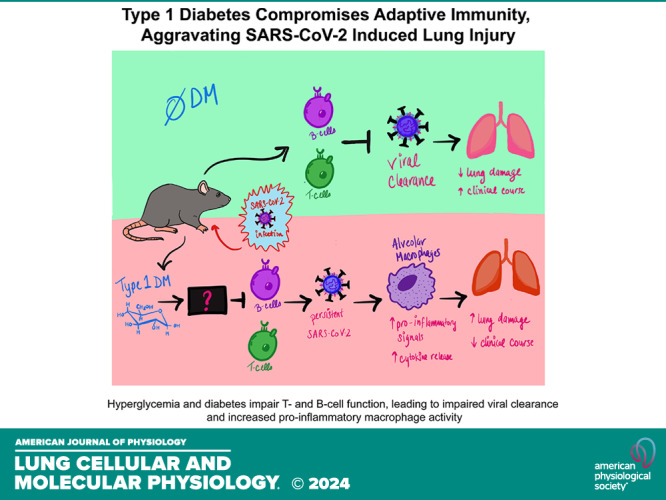

## INTRODUCTION

SARS-CoV-2 infection/COVID-19 commonly presents as pneumonia, with those most severely affected progressing to acute respiratory distress syndrome (ARDS), resulting in a mortality rate as high as 40% (1). Patient responses to SARS-CoV-2 infection are heterogeneous, ranging from asymptomatic to ARDS to initially mild disease later manifesting chronic sequelae (“long COVID”) (1, 2). At least some of this heterogeneity is due to patient risk factors and comorbidities, with metabolic diseases including type 1 and type 2 diabetes mellitus, as well as obesity and hyperlipidemia, significantly increasing the odds of severe disease (2–5). Despite numerous theories, the molecular basis by which these comorbidities promote disease progression remains unclear.

Type 1 diabetes mellitus (T1DM) is an autoimmune disease caused by the destruction of the beta cells of the pancreas responsible for making insulin (6). Although there has been a plethora of research into the underlying autoimmunity responsible for the onset of T1DM, the subsequent impact of hyperglycemia and the systemic diabetic phenotype on immune function remains understudied (7). Though studies in both type 1 and type 2 DM have shown that hyperglycemia itself has a negative impact on the immune system, it does not seem to be the only factor mediating this dysfunction (7, 8). In addition to COVID, patients with diabetes have been found to have worse outcomes with other respiratory viruses, such as influenza and respiratory syncytial virus (9, 10).

In this study, we sought to explore the effect of T1DM and hyperglycemia on the severity and course of SARS-CoV-2 infection. We utilize a well-established murine model of T1DM (11) and a mouse-adapted strain of SARS-CoV-2 known to induce significant pulmonary disease (12), and we observed that hyperglycemia does in fact confer a much more severe and morbid SARS-CoV-2 infection. Furthermore, through transcriptomic and cytometric analyses, we identified a critical deficiency in the adaptive immune system that likely explains at least some of the more deleterious effects of infection on the lungs of T1DM mice including impaired viral clearance. Employing single-cell transcriptomics, we demonstrate that upon infection, diabetic animals with impaired adaptive immunity exhibit an enhanced proinflammatory phenotype of alveolar macrophages that likely serve as a compensatory, if dysfunctional, response leading to increased morbidity. In addition, as a result of innovations necessary for the analysis of tissue from Animal Biosafety Level 3 (ABSL-3) conditions, we present here a novel adaptation of a protocol that allows for thorough and quantitative immunophenotypic analysis of fully fixed tissues, even those that had been previously embedded for cryosectioning. This technique will allow for greatly expanded analyses of fixed tissues broadly from high biosafety level experiments and archived samples.

## METHODS

### Mice

Wild-type C57BL/6 male mice (Jackson) were housed in the Animal Biosafety Level 1 facility at the University of Pennsylvania under the standard dark/light cycle, ambient temperature, humidity, and within the Allentown individual ventilated BCU2 caging system. Animals were 8 wk of age and littermates, randomly assigned to each group. All animal experiments were approved and carried out under the guidelines set by the University of Pennsylvania’s Institutional Animal Care and Use Committees and followed all National Institutes of Health (NIH) Office of Laboratory Animal Welfare regulations.

### Streptozotocin-Induced Type 1 Diabetes

Mice were housed in an Animal Biosafety Level 1 facility. Adult mice were randomly assigned to receive 150 mg/kg streptozotocin (STZ) in sodium citrate buffer or sodium citrate buffer as vehicle control via intraperitoneal injection (11) on *day 28* and were allowed to recover for 4 wk before further experimentation. Sucrose was added to their water (30% sucrose by volume) for 5 days postinjection to support possible hypoglycemia as a result of the pancreatic beta cells releasing stored insulin subsequent to their destruction. Blood glucose levels were checked on *days 18* and *7* after 6 h of fasting using tail vein blood samples and Contour Next EZ Blood Glucose Monitoring System. Mice who had received STZ but did not meet the criteria for hyperglycemia (fasting blood glucose >250 mg/dL) were excluded.

### Mouse-Adapted SARS-CoV-2 Injury

Mice were housed in the Animal Biosafety Level 3 facility at the University of Pennsylvania under the standard dark/light cycle, ambient temperature, humidity, and within the Allentown individual ventilated BCU2 caging system. All work was in adherence to the University of Pennsylvania’s approved ABSL-3 Institutional Biosafety Committee (IBC) and Institutional Animal Care and Use Committee (IACUC) protocols. For some experiments (whole lung bulk RNA sequencing, Fig. 2), mice were randomly assigned to one of two treatment groups: mock infected and infected. Mice were anesthetized with ketamine-xylazine and infected with 10^4^ plaque-forming units (PFU) of mouse-adapted SARS-CoV-2 (SARS2-N501Y_MA30_) intranasally in 40 µL DMEM. The virus was propagated from the MA_30_ stock virus (a generous gift from Stanley Perlman) in TMPRSS2-expressing Vero cells (12). The TMPRSS2 Vero cells were grown in Dulbecco’s modified Eagle’s medium DMEM, supplemented with 10% FBS. The virus was sequenced after propagation and found to match the input strain. Mice were monitored daily and scored for clinical disease, and body weight was measured. At the clinical or experimental endpoint (7 days postinfection), mice were anesthetized by isoflurane and necropsied. All tissues were inactivated and removed from the Animal Biosafety Level 3 facility in accordance with the University of Pennsylvania IBC protocol.

### Whole Lung Bulk RNA Sequencing

Lung tissue was placed in TRIzol reagent (Thermo Fisher, PN 15596018) RNA was extracted using Direct-zol RNA Miniprep Plus kit from Zymo. cDNA synthesis, sequencing library generation, and sequencing were performed on an Illumina HiSeq platform by GENEWIZ Co. Ltd. Raw data (raw reads) in fastq.gz format were processed through a general pipeline, as described previously (14). Reads were aligned to the mm10 mouse genome using Kallisto and imported into R Studio for analysis via the TxImport package. Data were then normalized using the trimmed mean of *M* values normalization method in the EdgeR package. Mean-variance trend fitting, linear modeling, and Bayesian statistics for differential gene expression analysis were performed using the Voom, LmFit, and eBayes functions, respectively, of the Limma package, yielding differentially expressed genes between uninfected, infected, euglycemic, and diabetic groups. Volcano plots were created using the OmicStudio tools at https://www.omicstudio.cn/tool. All detectable genes derived from RNA-seq were used for gene set enrichment analysis (GSEA) using the Molecular Signatures Database (MSigDB) C2: curated gene sets according to the standard GSEA user guide (http://www.broadinstitute.org/gsea/doc/GSEAUserGuideFrame.html).

### RNA Isolation and Quantitative PCR

Total RNA was extracted by Direct-zol RNA Miniprep Plus kit according to the manufacturer’s instruction (Zymo, PN R2072) and then reverse-transcribed into complementary DNA using the iScript Reverse Transcription Supermix (Bio-Rad, PN 1708841). Quantitative PCR (qPCR) was performed using a PowerUp SYBR Green Master Mix and standard protocols on an Applied Biosystems QuantStudio 6 Real-Time PCR System (Thermo Fisher Scientific). RPL19 was used to normalize RNA isolated from mouse samples. The 2^−ΔΔCt^ comparative method was used to analyze expression levels. The primers used are listed in Supplemental Table S1.

### Ex Vivo Alveolar Macrophage Experiments

Alveolar macrophages were isolated from wild type (WT), uninjured C57BL/6 mice using bronchial alveolar lavage (BAL) and expanded in culture media using previously described methods (15, 16). Mice were euthanized by isoflurane overdose and bronchoalveolar lavage fluid (BALF) was collected using a 20G angiocath through an incision in the trachea. Lungs were lavaged with a total of 10 mL BAL buffer (15, 16) warmed to 37°C, using 1 mL at a time. The collected BALF was stained with Brilliant Violet 421 anti-mouse F4/80 antibody (1:100; BioLegend, Clone BM8), and alveolar macrophages were sorted with a BD FACSAria Fusion Sorter (BD Biosciences). The cells were then expanded in RPMI 1640 containing 20 ng/mL murine granulocyte-macrophage-stimulating factor (GM-CSF, Peprotech PN 315-03) in a 12-well plate. Once all wells reached adequate expansion of cells, media was switched to that containing low glucose (base composition of glucose in RPMI 1640), high glucose (additional 50 mM glucose added to RPMI 1640 media), with additional conditional media containing low and high concentrations of polyinosinic:polycytidylic acid (poly I:C; 2 μg/mL and 10 μg/mL respectively; Bio-techne PN 4287/10) for 24 h before cells were harvested for RNA using the ProMega ReliaPrep RNA Miniprep System (PN Z6011). qPCR was performed using the protocol detailed previously, and primers used for gene expression determination are listed in Supplemental Table S1.

### Fixed Tissue Single-Cell Digest

Given the requirements for ABSL-3 safety precautions, the tissue harvested from these mice needs to be fixed in paraformaldehyde for at least 24 h prior to further handling. Logistically, the digestion of tissue into a single-cell suspension prior to the required fixation step was impossible due to time and staff constraints. To facilitate these experiments, we adapted a novel technique originally developed for the analysis of fixed tissue for scRNA-seq, Tissue Fixation & Dissociation for Chromium Fixed RNA Profiling (10x Genomics, Protocol CG000553). Tissue was harvested in the ABSL-3 facility by trained personnel and minced into square millimeter sized pieces before being fixed for 24 h at 4°C. Samples dedicated to scRNA sequencing were fixed with Fix & Perm Buffer (10x Genomics, PN-2000517) as per manufacturer’s protocol while samples for flow cytometry were fixed in 4% paraformaldehyde (PFA). Samples were then washed with PBS and the fixation reaction was quenched. scRNA samples were quenched with Quench Buffer (10x Genomics, PN-2000516) as per the manufacturer’s protocol, while flow cytometry samples were quenched in the solution of 50 mM glycine dissolved in PBS. Tissue was then dissociated into a single-cell suspension shaking vigorously at 37°C for 1 h in a dissociation solution made with Liberase TL (concentration 0.2 mg/mL, MilliporeSigma, PN 5401020001) as per the manufacturer’s protocol. Tissue was further triturated with a P1000 pipette tip 15–20 times before being filtered through a 40 µM filter, which was then washed with PBS. The samples were quenched again with Quench Buffer and cells were then counted by hemocytometer before proceeding to scRNA sequencing or flow cytometry. Samples were stored at 4°C for up to 1 wk before use. Samples designated for scRNA sequencing were incubated in a Quenching Buffer and Enhancer (10x Genomics, PN-2000482) as per the manufacturer’s protocol.

### Histology

Lung tissues were fixed in 4% PFA for 24 h at room temperature in accordance with the ABSL-3 safety protocols, rinsed three times with PBS at room temperature, incubated in 30% sucrose overnight at 4°C, and transferred to 15% sucrose 50% optimal cutting temperature (OCT) compound at room temperature for 4 h. Fixed tissues were transferred to an embedding mold filled with OCT compound, frozen on dry ice, and stored at −80°C. Frozen sections (7 µm thick) were cut at −20°C, and slides were kept at −20°C until use.

### Hematoxylin and Eosin Staining and Immunofluorescence

Sections were stained with a Hematoxylin and Eosin Stain Kit (Vector Laboratories, PN H-3502) according to the manufacturer’s protocol and then imaged with a Leica DMi8 microscope. For immunohistochemical analysis, the slides were postfixed for another 5 min with 3.2% PFA. Tissue sections were blocked in blocking buffer (2% BSA, 5% donkey serum, and 0.1% Triton X-100 in PBS) for 1 h at room temperature. Afterward, slides were probed with primary antibodies for T- and B-cells (PE/Cyanine5 anti-mouse CD3ε antibody 1:200, BioLegend, Clone 145-2C11 and FITC anti-mouse/human CD45R/B220 antibody 1:200, BioLegend, Clone RA3-6B2, respectively) and incubated overnight at 4°C. The next day, the slides were washed and incubated with the fluorophore-conjugated secondary antibodies (typically Alexa Fluor conjugates, Life Sciences) at a 1:1,000 dilution for 1 h. Last, slides were again washed, incubated with 1 µM 4′,6-diamidino-2-phenylindole (DAPI) for 5 min, and mounted using ProLong Gold (Life Sciences, PN P36930). Four to six images were taken randomly from each sample/section with a Leica DMi8 microscope.

### Fluorescence Activated Cell Analysis

Whole lung single-cell suspensions from mice on *day 7* post-MA_30_SARS-CoV2 infected lungs were prepared as above. Single-cell suspensions were blocked in FACS buffer containing 1:50 mouse TruStain FcX for 10 min at room temperature. The cell suspension was stained using Brilliant Violet 785 anti-mouse CD45 antibody (1:100, BioLegend, Clone 30-F11), Brilliant Violet 421 anti-mouse F4/80 antibody (1:100; BioLegend, Clone BM8), PE/Cyanine5 anti-mouse CD3ε antibody (1:200, BioLegend, Clone 145-2C11), Brilliant Violet 785 anti-mouse CD4 antibody (1:50, BioLegend, Clone GK1.5), APC anti-mouse CD8a Recombinant antibody (1:100, BioLegend, Clone QA17A07), PE anti-mouse Ly-6G antibody (1:200; BioLegend, Clone 1A8), PE/Cyanine7 anti-mouse CD64 (FcγRI) antibody (1:200, BioLegend, Clone X54-5/7.1), Brilliant Violet 711 anti-mouse/human CD11b antibody (1:200; BioLegend, Clone M1/70), and FITC anti-mouse/human CD45R/B220 antibody (1:200, BioLegend, Clone RA3-6B2) overnight at 4°C. Stained cells and “fluorescence minus one” (FMO) controls were then resuspended in FACS buffer. All flow analyses were performed on BD LSRFortessa (BD Biosciences). For FACS analysis of uninfected DM and non-DM mouse lungs, single-cell suspensions were prepared through more traditional methods. Briefly, the lungs were thoroughly perfused with cold PBS via the left atrium to remove residual blood in the vasculature. Lung lobes were separated, collected, and digested with collagenase type II (5 mg/mL in HBSS) (Worthington Biochemical, LS004176) for 1 h at 37°C in a shaker at a speed of 70 rpm. They were then mechanically dissociated in a sort buffer (DMEM + 2% Cosmic Calf Serum + 2% Penicillin/Streptomyocin). Next, cell suspensions were treated by red blood cell lysis buffer containing 1:1,000 deoxyribonuclease I (DNase I) (MilliporeSigma, PN D4527) for 5 min at room temperature and were filtered through a 70-µm cell strainer. They were then stained as described earlier and analyzed on a BD LSRFortessa.

### Single-Cell RNA Sequencing Library Construction

Whole lung single-cell suspensions from mice on *day 7* post-MA_30_SARS-CoV2 infected lungs were prepared as above and were used for sequencing. Single-cell sequencing libraries were created according to 10x Genomics Chromium Fixed RNA Profiling Reagents (10x Genomics, PN 1000414) and associated user protocol (User Guide CG000477 Rev D). After fixation, the samples were first hybridized using mouse-specific whole transcriptome probe pairs. Cell counts were verified using a LogosBio Luna-FL automated cell counter (1.05e6 cells·mL^−1^ and 2.8e6 cells·mL^−1^ for samples 708 L and 719 L, respectively). The cells were pelletized and re-suspended in a hybridization mixture prepared using 10x Genomics Hyb Buffer B (PN 2000483) and Enhancer (PN 2000482) according to the protocol. Mouse-specific WTA Probes BC001 (10x Genomics, PN 2000703) were then added to each sample at room temperature. The samples were incubated at 42°C in a Bio-Rad C1000 Touch Thermal Cycler for 16 h.

Following probe hybridization, each sample was washed in a Post-Hyb Wash Buffer prepared according to the protocol using concentrated Post-Hyb Buffer (PN 2000533) and Enhancer (PN 2000482). The samples were then incubated at 42°C, centrifuged for 5 min at 850 relative centrifugal force (rcf), and resuspended in a Post-Hyb Buffer. This wash procedure was repeated for a total of three washes. Each sample was then filtered through a 30 µm Sysmex CellTrics filter into a clean 1.5 mL LoBind Eppendorf Tube. The cell concentration was then verified again using the LogosBio Luna-FL automated cell counter according to the manufacturer’s protocol (3.86e6 cells·mL^−1^ and 1.59e6 cells·mL^−1^ for sample 708 L and 719 L, respectively). Gel Beads-in-Emulsion (GEMs) were then generated using the 10x Next GEM Chip Q according to the manufacturer’s protocol and loaded onto a 10x Genomics Chromium X Chip Reader. Cells were loaded onto the chip targeting a total of 10,000 cells per sample. Upon completion of the chip being processed, the GEMs were transferred to new PCR strip tubes and incubated in a Bio-Rad C1000 Thermal Cycler according to the protocol. The samples were left in a 4°C refrigeration overnight.

The samples were then mixed with 10x Genomics proprietary recovery agent (PN 220016). The recovery agent is then removed and discarded after 2 min from the resulting biphasic mixture. The remaining aqueous phase is then amplified according to the manufacturer’s protocol. A DNA cleanup is then performed on the amplified DNA using Beckman Coulter SPRIselect magnetic beads. Gene Expression libraries were then created according to the protocol, using 12 cycles of the appropriate PCR program supplied by the manufacturer. The samples then underwent another cleanup using SPRIselect magnetic beads. Libraries were quantified using the Invitrogen High Sensitivity DNA Qubit Assay and library quality was checked using the Agilent Tapestation 4200 controller and High Sensitivity D1000 Assay. The libraries were sequenced on an Illumina NextSeq 2000 using a P2 100 Cycle kit. A 2.05 nM pool was prepared, and the sequencing cartridge was loaded at 650 pM with 1 µL of 1 nM PhiX. A minimum of 10,000 read pairs were targeted for each sample with paired-end dual indexing. The parameters for each read were 28 cycles for Read 1, 10 cycles for the i7 index, 10 cycles for the i5 index, and 90 cycles for Read 2, per the protocol.

### Single-Cell Transcriptomics

After sequencing, initial data processing was performed using Cellranger (v7.2.0). Cellranger mkfastq was used to generate demultiplexed FASTQ files from the raw sequencing data. Next, Cellranger count was used to align sequencing reads to the mouse reference genome (GRCm38) and generate single-cell gene barcode matrices. Post-processing and secondary analysis were performed using the Seurat package (v.4.0) (17). First, variable features across single cells in the dataset were identified by mean expression and dispersion. Identified variable features were then used to perform a principal component analysis (PCA). The dimensionally reduced data were used to cluster cells and visualize using a uniform manifold approximation and projection (UMAP) plot (18). Cell populations were identified by comparison to known marker genes for each cell type. Differential expression analysis between samples and populations within samples was performed using the default variable with the command FindMarkers.

### Statistics

All statistical calculations were performed using GraphPad Prism 9. Unpaired two-tailed Student’s *t* tests were used to ascertain statistical significance between the two groups. For details on statistical analyses, tests used, size of *n*, definition of significance, and summaries of statistical outputs, see the corresponding figure legend and results.

## RESULTS

### Hyperglycemia Renders Mice More Susceptible to Severe SARS-CoV-2 Infection and Impairs Recovery

**Figure 1.**
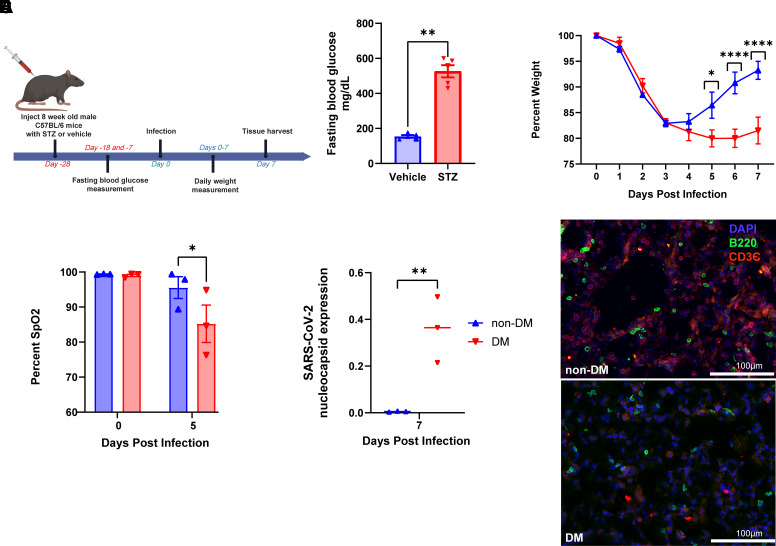
Hyperglycemia and DM render male mice more susceptible to severe SARS-CoV-2 infection. *A*: schematic of experimental design for inducing T1DM in wild-type mice. Figure made with BioRender. *B*: STZ effectively creates fasting hyperglycemia in wild-type mice. *C*: time course of changes in body weight in male non-DM (*n* = 7) and DM (*n* = 4) mice after MA_30_SARS-CoV-2 infection. *D*: comparison of capillary oxygen saturation of male non-DM and DM mice at *day 0* (uninjured) and *day 5* postinfection, *n* = 3 per group. *E*: qPCR analysis of SARS-CoV-2 nucleocapsid protein expression in lungs of male DM and non-DM mice harvested 7 days postinfection, *n* = 3 per group. *F*: representative B220/CD3 stained sections of lung tissue harvested from male non-DM and DM mice 7 days postinfection with MA_30_SARS-CoV-2, scale bars 100 µm. Data in *B*, *C*, *D*, and *E* are presented as means ± SE, calculated using one-way ANOVA, followed by Mann–Whitney test (*B*), Sidak’s multiple comparison test (*C*), uncorrected Fisher’s LSD (*D*), and Tukey’s multiple comparison test (*E*). ANOVA, analysis of variance; LSD, least significant difference test; qPCR, quantitative PCR; STZ, streptozotocin; T1DM, type 1 diabetes mellitus. *P < 0.05, **P < 0.01, and ****P < 0.0001.

The utilization of high-dose streptozotocin (STZ) is an effective and widely used technique for inducing T1DM in mice (11). This alkylating agent operates by selectively binding to and inducing toxicity of the pancreatic β-cells, which are responsible for insulin production. Consequently, this model closely replicates the pathophysiology of T1DM, though we note that it is not thought to recapitulate the autoimmune aspects of the disease. We treated wild-type C57BL/6 mice with a single, high dose of STZ (Fig. 1*A*), which resulted in the intended outcome of hyperglycemia after 10 days (Fig. 1*B*). We then subjected hyperglycemic and vehicle-treated, euglycemic control mice to infection with MA_30_SARS-CoV-2, a mouse-adapted strain recognized for inducing significant pulmonary disease. Specifically, T1DM-afflicted mice exhibited more pronounced weight loss, exacerbated oxygenation deficits, and a slower recovery trajectory in comparison with their euglycemic counterparts (Fig. 1, *C* and *D*). Notably, hyperglycemia was preserved in T1DM animals at *day 7* postinfection (Supplemental Fig. S1). Tissue damage was apparent in the lungs after infection as judged by hematoxylin and eosin staining (Supplemental Fig. S2). Although these outcomes were apparent in both sexes, the differences were more stark in males (Supplemental Fig. S3), so we utilized males for subsequent experiments unless otherwise noted. We performed qPCR analysis of RNA extracted from the whole lungs of both DM and non-DM mice at *day 7* postinfection to assess the level of SARS-CoV-2 nucleocapsid protein transcript as a substitute for viral plaque assays given the restrictions of ABSL-3 safety precautions. Critically, we noted that T1DM mice were markedly less effective in clearing the virus by the seventh day postinfection, while euglycemic mice had essentially undetectable viral loads by this time point (Fig. 1*E*). We also assessed common cytokine expression in these samples, noting IL-6 is significantly upregulated in male DM lungs at *day 4* postinfection and Cxcl9 is downregulated in DM lungs at *day 7* postinfection, but no other apparent differences (Supplemental Fig. S4). Intriguingly, and in contrast to recently published work (19), we did not observe any overt phenotype upon H1N1 influenza infection in these mice (Supplemental Fig. S5). These findings not only emphasize the impact of diabetes on the severity of SARS-CoV-2 infection but also underscore the intricate interplay between metabolic disorders and viral pathogenesis and indicate that at least some components of increased morbidity of T1DM subjects result from an impaired ability to effectively clear SARS-CoV-2 virus.

### Whole Lung RNA Sequencing Reveals Deficiency in Adaptive Immune Response in Diabetic Mice as a Potential Etiology for Prolonged Morbidity and Impaired Recovery

**Figure 2.**
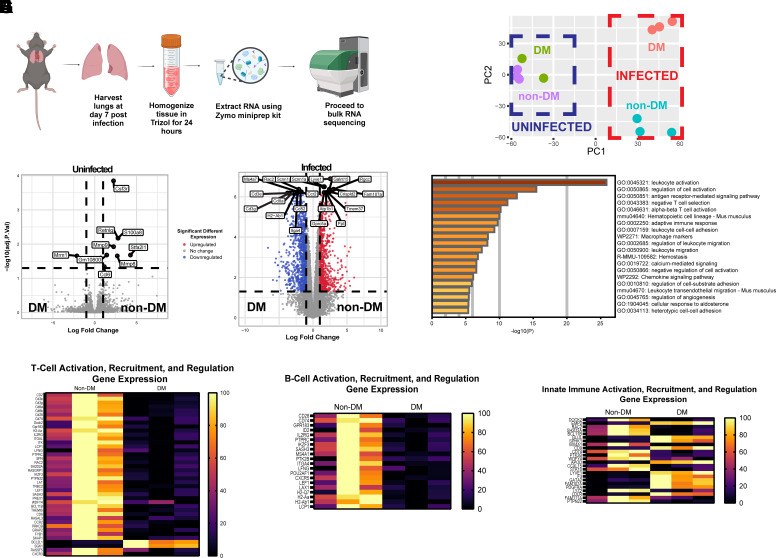
Whole lung RNA sequencing reveals an impaired adaptive immune response in male DM mice infected with MA_30_SARS-CoV-2. *A*: schematic for experimental design of whole lung RNA sequencing. Figure made with BioRender. *B*: PCA plot of whole lung RNA sequencing of tissue harvested from male non-DM, uninfected mice, DM, uninfected mice, non-DM, infected mice, and DM, infected mice, *n* =3 per group. *C* and *D*: volcano plots of significantly differentially expressed genes in the lungs of uninfected non-DM and DM mice (differential gene expression meeting significance = 9 genes; *C*) and in the lungs of infected non-DM and DM mice (differential gene expression meeting significance = 1,745 genes; *D*). *E*: the GO terms of differentially expressed genes in non-DM and DM mice lungs infected with MA_30_-SARS-CoV-2 were carried out by Metascape (13). *F–H*: heatmap showing the top differentially expressed genes between non-DM and DM mice in relation to the activation, recruitment, and regulation of T-cells (*F*), B-cells (*G*), and innate immune cells (*H*). GO, Gene Ontology; PCA, principal component analysis.

We sought to take an unbiased approach to hypothesis generation for underlying mechanisms to explain the differences in clinical course and viral clearance observed in T1DM animals. We thus employed whole lung, “bulk” RNA sequencing to further examine the transcriptomic changes. Importantly, while there were only minimal changes (9 genes) present between the two groups of uninfected mice, upon infection the number of differentially expressed genes increased sharply to >1,500 (Fig. 2, *C* and *D*), reinforcing the large changes in diabetic host response upon MA-SARS-CoV-2 infection. Further examination of the data highlighted a significant downregulation of genes specifically associated with the adaptive immune system in the lungs of DM mice, as illustrated in Fig. 2*E*. This downregulation extended to genes crucial for the activation, recruitment, and proliferation of both T-cells (Fig. 2*F*) and B-cells (Fig. 2*G*). Interestingly, many genes associated with innate immunity were also differentially expressed, though with no clear pattern weighted toward either group of infected mice. Taken together, these results indicate that hyperglycemia renders mice more susceptible to severe disease with MA_30_SARS-CoV-2, and that impaired adaptive immunity appears to explain many components of the observed phenotype including the persistence of viral particles and relative downregulation of adaptive immune gene markers in STZ-treated animals upon infection.

### Development of a Novel Flow Cytometric Immunophenotyping Strategy for Fixed Tissue Corroborates Impaired Adaptive Immunity in Diabetic Animals

**Figure 3.**
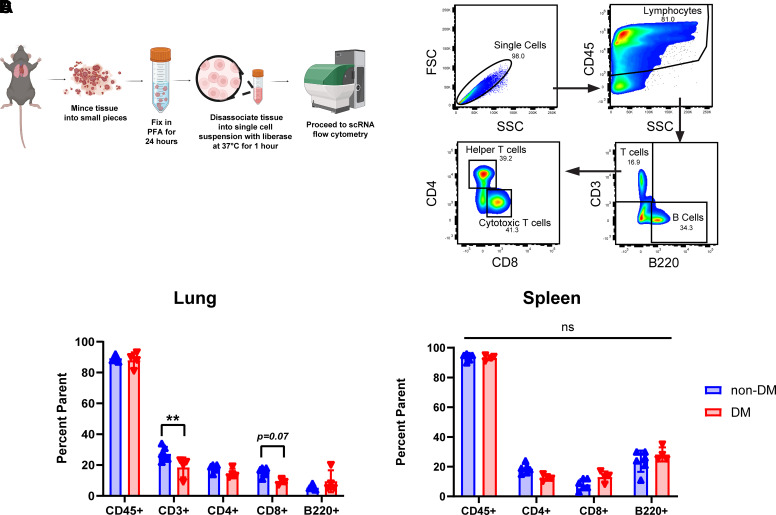
Novel technique for single-cell digestion of fixed tissue and flow cytometric analysis. *A*: schematic of protocol for digesting fixed tissue from ABSL-3 facility into single-cell digest for flow cytometry analysis. Figure made with BioRender. *B*: representative gating scheme for identification of lymphocytes (CD45^+^), all T-cells (CD3^+^), including helper T-cells (CD4^+^) and cytotoxic T-cells (CD8^+^), and B-cells (B220^+^) by flow cytometry in the lungs and spleens of non-DM and DM mice infected with MA_30_SARS-CoV-2. *C* and *D*: quantification of the proportion of different immune populations in the lungs (*C*) and spleens (*D*) of male non-DM and DM mice at 7 days postinfection. Data in *C* and *D* are presented as means ± SE, calculated using one-way ANOVA, followed by Sidak’s multiple comparison test. ABSL-3, Animal Biosafety Level 3; ANOVA, analysis of variance. **P < 0.01.

One of the challenges of studying a pathogen as virulent as SARS-CoV-2 is the requirement for ABSL-3 precautions. As such, sample neutralization by fixation or heat inactivation is required for biosafety reasons, leaving extremely limited options for downstream analysis of biological materials. We therefore adapted a method of dissociating fixed tissue into a single-cell solution, originally developed for single-cell RNA sequencing (Fig. 3*A*) (20). We have validated that this methodology works well with flow-based immunophenotyping of common markers including CD45, CD4, CD8, and B220 (Fig. 3*B*), though we note that given variability in tissue digest, we have greater confidence in percentage parent values rather than total cell numbers using this protocol. Excitingly, we also demonstrated that this method also works well even with tissue cryoembedded in OCT (Supplemental Fig. S6, methods).

We then went about performing flow cytometry to better characterize the differences in immune response between the hyperglycemic and euglycemic cohorts. In addition to looking at immune cell numbers from the lungs of these mice, we also compared the immunoprofile of the spleens as a representative of the peripheral immune system to assess whether treatment with STZ in any way “primed” the immune system prior to SARS-CoV-2 infection that may explain the findings from whole lung RNA seq. In addition, several studies have demonstrated the immune modifying effects of STZ, particularly in T-cells (21), so we also performed immunophenotyping of the lungs of STZ and vehicle-treated mice that were not infected with MA-SARS-CoV-2. Focusing especially on changes in B- and T-cells as suggested by our RNA-Seq findings, this approach allowed us to utilize traditional FACS-based methods to validate transcriptomic observations. This approach revealed a significant decrease in the CD3^+^ T-cell population in the lungs of DM mice as compared with their euglycemic cohort and a nearly significant decrease specifically in CD8^+^ T-cells (Fig. 3*C*), consistent with the results of the whole lung bulk-RNA seq experiment. We also corroborated this by tissue section staining (Fig. 1*F*). There was however no significant difference in the % of CD45^+^, B220^+^, CD4^+^, and CD8^+^ cells in the spleens of the two infected groups (Fig. 3*D*), nor in the uninfected lungs of the two cohorts (Supplemental Fig. S7), suggesting that STZ has no obvious effect on the peripheral adaptive immune system composition prior to pathogen challenge.

### Single-Cell Transcriptomic Analysis Reveals a Distinct Macrophage Population in the Lungs of Diabetic Mice post-SARS-CoV-2 Infection

**Figure 4.**
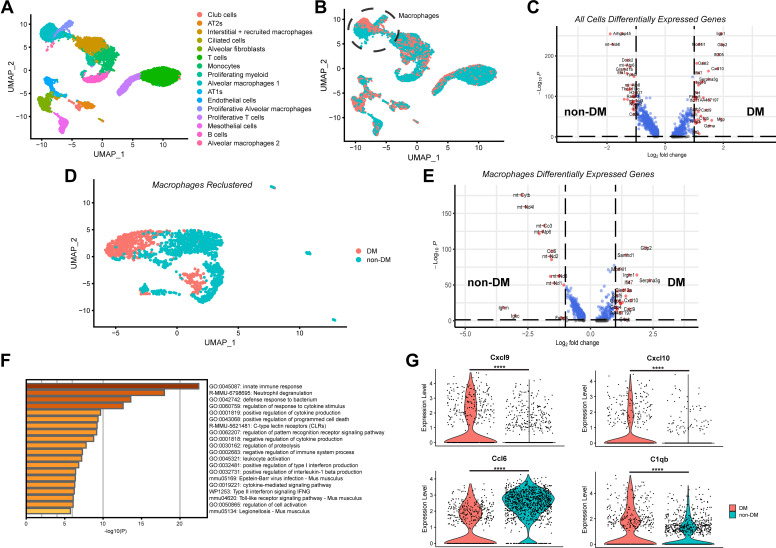
Single-cell transcriptomic analysis reveals a distinct macrophage population in the lungs of male DM mice post-SARS-CoV2 infection. *A*: scRNA-seq analysis for lungs harvested from male non-DM and DM mice infected with MA_30_SARS-CoV-2 at *day 7* postinfection, cells are color coded for population clustering and presented in UMAP plot. *B*: UMAP plot from *A* with cells color coded by sample of origin (non-DM vs. DM). *C*: volcano plot of significantly differentially expressed genes in the lungs of non-DM and DM mice at *day 7* postinfection (differential gene expression meeting significance = 1,996 genes). *D*: UMAP plot configured from a subset of *A* looking at macrophages with cells color coded by sample of origin (non-DM vs. DM). *E*: volcano plot of significantly differentially expressed genes in the alveolar macrophages of non-DM and DM mice at *day 7* postinfection (differential gene expression meeting significance = 1,875 genes). *F*: the GO terms of differentially expressed genes in alveolar macrophages of non-DM and DM mice lungs infected with MA_30_SARS-CoV-2 reveals DM macrophages upregulate genes responsible for inflammation. *G*: violin plots of cytokine genes in alveolar macrophages compared between non-DM and DM mice lungs infected with MA_30_SARS-CoV-2. All comparisons shown in *G* have an adjusted *P* value of < 5 × 10^−4^ using Wilcoxon rank sum test within Seurat. **** *P* value < 1 × 10^−17^. UMAP, uniform manifold approximation and projection.

Recognizing that bulk RNA-Seq suffers from potential obfuscation of discrete signals driving the antiviral response from a rare cell population or cell type, we sought to better characterize the effects of hyperglycemia and T1DM on the pulmonary host response to SARS-CoV-2 by performing single-cell RNA sequencing (scRNA seq) on lungs harvested from mice infected with SARS-CoV-2. Single-cell suspensions were created from the fixed lung tissue of STZ- and vehicle-treated mice infected with SARS-CoV-2 at *day 7* postinfection as in our flow cytometry experiments. Libraries were created using the 10x Genomics Chromium Fixed RNA Profiling kit and sequenced on an Illumina NextSeq 2000. A total of 10,726 cells were sequenced with 3,197 cells from DM lungs and 7,529 cells from non-DM lungs. Cluster analysis in Seurat identified 16 separate and distinct clusters of cell types based on marker gene expression (Fig. 4*A*). These 16 clusters include subtypes of cells found in the epithelial, mesenchymal, endothelial, and immune compartments of the lung, as was expected. Analysis of the clusters in the uniform manifold approximation and projection (UMAP) by cells labeled by sample of origin reveals a clear reduction in number of cells involved in adaptative immunity in the lungs of the DM mice (Fig. 4*B*). In particular, the T- and B-cell clusters are overwhelmingly represented by cells from the non-DM sample. Alveolar fibroblasts seem to be more prevalent in the lungs of DM mice, possibly as a result of increased inflammation and inflammatory signals from the persistence of viral particles.

Notably, we observed a clear difference in alveolar macrophages (AMs) between the two samples (Fig. 4*B*, circle) that did not appear to simply be a reflection of differential cell numbers. Instead, the transcriptional differences between the AMs of the two groups were apparent in the separation in clustering on the UMAP. These differences were emphasized upon isolation and reclustering of the macrophages (Fig. 4*D*), 5w?>where the AMs from DM mouse lungs showed almost no spatial overlap with those from non-DM mice. The transcriptional differences driving this separation are highlighted in volcano plots of significantly differentially expressed genes between these two macrophage populations (Fig. 4*E*). Metascape analysis of the 200 most significantly upregulated genes in the DM macrophages revealed that a majority of these genes that are upregulated are involved in cytokine release and response, pro-inflammatory signals, and programmed cell death (Fig. 4, *F* and *G*). This heightened inflammatory state corroborates the increased weight loss and delayed recovery the DM mice experience with SARS-CoV-2 infection and may be a compensatory, yet dysregulated, response to the deficient adaptive immune response as evidenced by the lower number of adaptive immune cells seen both by flow cytometry and scRNA seq. The same reclustering approach was done with T-cells to investigate any significant differences between the populations other than just differential abundance (Fig. 4*B*). The resulting UMAP corroborated the results observed from the larger cell subset, which is that there is significant spatial overlap between the T-cells from both cohorts (Supplemental Fig. S8*A*). One portion of the projection that seemed to have reasonable separation of cells based on the sample of origin was proliferative T-cells (Supplemental Fig. S8*B*). This reclustering made more apparent the decrease in CD8^+^ and CD4^+^ cells in the DM sample, compared with the non-DM lungs (Supplemental Fig. S8, *C* and *D*).

Taken together, these findings suggest a model in which T1DM/hyperglycemia causes adaptive immune defects (especially in T-cell activation/recruitment), resulting in alveolar macrophages adopting a compensatory proinflammatory phenotype and contributing to greater clinical symptoms of the disease.

### Viral Mimics “Reprogram” Wild-Type AMs in Cell Culture to Transcriptionally Simulate AMs Found in the Lungs of DM Mice Infected with MA-SARS-CoV-2

**Figure 5.**
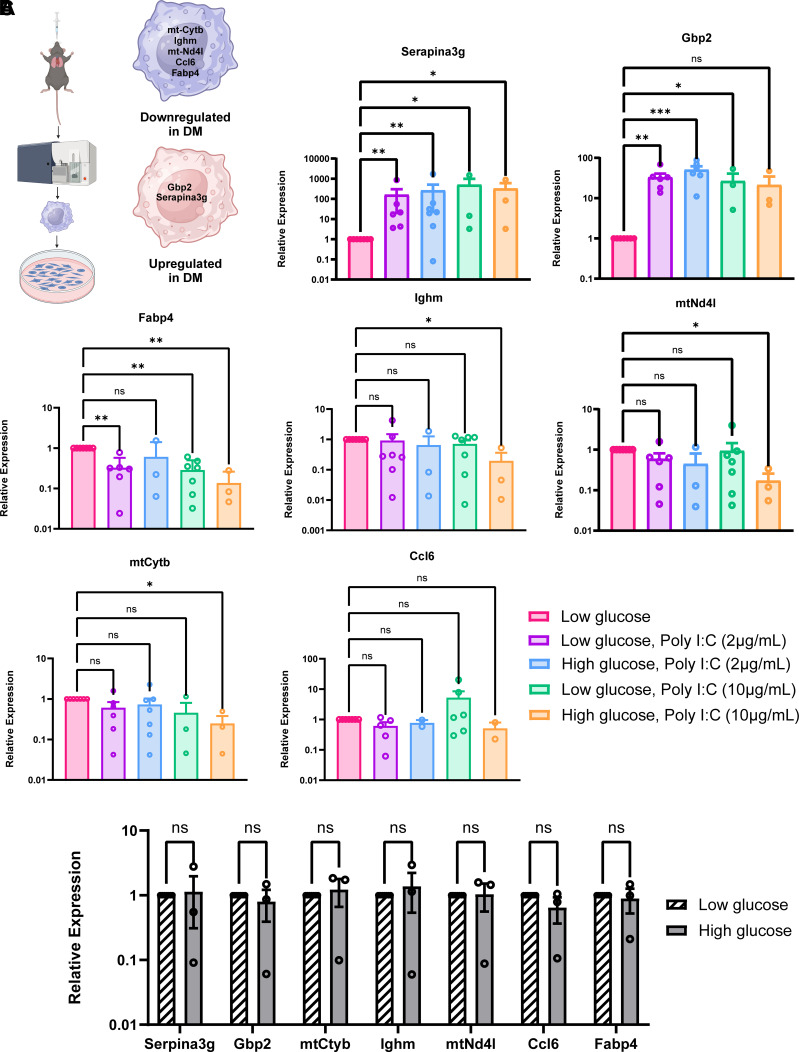
Viral mimics “reprogram” WT AMs in cell culture to transcriptionally resemble those found in the lungs of male DM mice infected with MA-SARS-CoV-2, whereas varying glucose concentration did not similarly affect gene expression. *A*: schematic of protocol for AM extraction from WT mice via BAL and FACS sorting. AMs were cultured in conditional media to reproduce the environment experienced by AM in vivo in DM mice infected with MA-SARS-CoV-2. Cells were harvested after 24 h and analyzed for gene expression levels using qPCR looking at the top differentially expressed genes found in the scRNA seq experiment (Fig. 4*E*). Figure made with BioRender. *B: Gbp2* and *Serpina3g* were significantly upregulated upon treatment with poly I:C whereas *Fabp4, Ighm*, *mt-Cytb*, and *mt-Nd4l* were all highly downregulated upon treatment with poly I:C. *C*: modulating glucose concentrations had no significant effect on the expression levels of any of these genes. Data in *B* and *C* are presented as means ± SE, calculated using one-way ANOVA, followed by uncorrected Dunn’s test. AMs, alveolar macrophages; ANOVA, analysis of variance; BAL, bronchial alveolar lavage; qPCR, quantitative PCR; WT, wild type. **P < 0.01.

To begin to address the underlying mechanisms driving the observed changes in macrophages in infected DM mice, we attempted to model the virulent diabetic microenvironment in vitro. Primary murine alveolar macrophages were isolated and treated 24 h with various levels of glucose (mimicking higher blood glucose in DM mice) and poly I:C (imitating the persistence of viral RNA seen in our infected DM cohort). We picked several of the top up- and downregulated genes from our scSeq data to address changes in response to these two variables (Fig. 5*A*). Two of the proinflammatory genes most highly enriched in DM macrophages (*Gbp2* and *Serpina3g*) were profoundly upregulated upon treatment with poly I:C, but modulating glucose levels had no significant impact (Fig. 5, *B* and *C*). Several metabolism-related genes were downregulated in DM macrophages (*Fabp4, Ighm*, *mt-Cytb*, and *mt-Nd4l*); these genes showed variable responses to glucose levels but were all highly downregulated upon treatment with poly I:C regardless of glucose levels. Taken together, our model suggests that the proinflammatory changes observed in alveolar macrophages in the lungs of SARS-CoV-2 infected DM mice are driven primarily by the inability to effectively clear virus rather than by hyperglycemia itself.

## DISCUSSION

Despite the start of the pandemic being December 2019 and the US government ending the federal Public Health Emergency for COVID-19 status (22–25), as of January 2024, more than 1000 Americans died from COVID per week (26)—not including those who have died from COVID-related long-term health detriments. COVID-19 thus clearly remains a major health concern. Patient responses to SARS-CoV-2 infection are varied, with patient comorbidities acting as major contributors to varied outcomes. Focusing on one such major comorbidity, we assessed whether pharmacological induction of T1DM would increase the severity of lung injury in a murine model of COVID-19 pneumonia. In spite of the multitude of studies looking at host response to SARS-CoV-2 since the pandemic began in 2019 (27–29), to our knowledge no existing studies have examined T1DM as a co-morbidity specifically with SARS-CoV-2 mouse models. Our findings indicate that T1DM mice exhibit deficiency in the recruitment of adaptive immune cells, resulting in impaired viral clearance and a dysregulated, pro-inflammatory response of the innate immune system, specifically within alveolar macrophages.

It is a widely held belief that people with DM are more vulnerable to infectious pathogens (30–32). Although there have been no conclusive studies defining mechanisms behind this phenomenon—and in all likelihood, such mechanisms are multifactorial and rely equally on both the pathogen and host status—several hypotheses have been explored (8, 32–34). Differential pathogenesis may result from the hyperglycemic environment altering antigen recognition sites (e.g., surface protein glycosylation) of pathogens (7, 8), allowing them to evade immune recognition. More recent studies have shown that glucose metabolism is critical to the function of innate immune cells in the lung and that the hyperglycemic state in patients with DM upsets this delicate balance, leaving the host more vulnerable to infections with influenza A in particular (19). Our findings recapitulate the notion that hyperglycemia and DM alter the function of the adaptive immune response with a significant deficiency in the recruitment and likely function of T- and B-cells but also revealed a pro-inflammatory innate immune profile compared with the euglycemic controls. Single-cell transcriptomics revealed that this proinflammatory switch was particularly apparent in alveolar macrophages. We postulate that this phenotypic change in macrophages represents a compensatory mechanism in response to impaired viral clearance by the diminished adaptive immune response that seems to be independent of the glucose concentration of their environment, instead driven by the persistence of viral particles as supported by our in vitro macrophage “reprograming” using poly I:C. Notably, the fundamental mechanism by which T1DM impairs adaptive immunity will require additional research, but given the stark differences in clinical course, our work highlights the critical importance of further elucidating how T1DM and hyperglycemia so profoundly disrupt adaptive immune responses. It is important to reiterate of course that mouse models of T1DM are imperfect and STZ treatment likely does not recapitulate the autoimmune drivers of T1DM in patients, pointing to the need for better models to fully model both the hyperglycemic and autoimmune components of the disease.

We must note that in contrast to recently published work (19), we did not observe a differential course of clinical disease upon H1N1 infection of T1DM animals, whereas the differences were stark and highly replicable with MA_30_SARS-CoV-2 infection. Many possible reasons for this discrepancy are possible. First, there could be an outsized role of the microbiome in these responses; the gut flora is known to be highly dependent on animal housing conditions, which often differs greatly between institutions. Differential dosing with virus or STZ may also make an impact. Furthermore, while DCs are not highly represented in our single-cell data, we did not note obvious transcriptomic differences in these cells between DM and non-DM mice upon SARS-CoV-2 infection. Future work should explore these differences.

Our study has shown that, in addition to a diminished adaptive immune response, alveolar macrophages in the infected lungs of DM animals are more inflammatory compared with those in euglycemic counterparts. We posit that this is a compensatory, if ineffective, reaction to persistence of virus, but this does contradict another widely held belief that DM is in fact protective against the development of ARDS (35–37). This prior argument posits that DM decreases the release of cytokines and interleukins, resulting in less chemotaxis and inflammation, which minimizes the risk of the dysregulated ARDS response that leads to leaky capillaries, dysfunctional epithelium, impaired gas exchange, and eventual death (35, 37). Our findings in this model of SARS-CoV-2 infection in T1DM mice directly contradict those hypotheses and appear supported by more recent clinical data indicating that patients with T1DM and T2DM were nearly three times more likely to die in the hospital compared with their age-matched peers at the beginning of the pandemic (4, 5).

In addition to the scientific advancements this study has provided, we highlight several methodological advances. Our adaptation of a technique that allows for the digestion of fixed tissue into a single-cell suspension that can be used for flow cytometry should enable expanded investigation of potentially lethal pathogens, which are otherwise hindered by the requirements of high biosafety level containment. Furthermore, the ability to disaggregate fixed tissue that has been embedded (in OCT or other materials) enables the study of banked tissue specimens, opening up a range of possibilities for further inquiry with previously disregarded samples.

Although we believe this work advances our knowledge as to how T1DM acts as a serious comorbidity for COVID-19, we acknowledge several limitations. We note that throughout this report, we have used “T1DM” and “DM” to denote animals treated with STZ that developed hyperglycemia. Although STZ is a well-established and widely accepted model of T1DM, we acknowledge that it does not capture the autoimmune pathophysiology of human disease and thus is not a perfect representation of patients with T1DM experiencing SARS-CoV-2 infection. In addition, we denote hyperglycemic animals as “DM” and use the terms interchangeably, acknowledging that our model more accurately reflects uncontrolled T1DM exhibited by hyperglycemia. Finally, due to the challenges of cohort size and reproducibility in ABSL3 facilities, we chose to focus on male mice since they exhibited more severe and reproducible responses to MA_30_SARS-CoV-2 infection. This highlights important and biologically interesting sex differences that are themselves worth exploring in future work.

T1DM is a disease that largely affects children and pneumonia is the number one cause of hospitalization in children less than 5 yr of age, with respiratory viruses accounting for up to 66% of those cases (9). Although we did not study T2DM in this study, T2DM has been identified as a serious global health threat by the World Health Organization, and the worldwide prevalence of T2DM is estimated to increase to 12.2% by 2040 (38). Given the new age of global viral pandemics and the increased morbidity and mortality patients with diabetes have already experienced with COVID, we can anticipate there will be future threats to the health and safety of these people in the coming decades. Further studies into the interplay between DM and immune dysfunction will uncover underlying mechanisms by which we can better adapt our treatment of an ever-growing patient population. Our results herald significant expansions in the understanding of how DM renders patients more vulnerable to an ever-present, evolving deadly threat and these findings have high potential to define new therapeutic targets to decrease viral pneumonia/ARDS severity and increase survival in these patients.

## DATA AVAILABILITY

Single-cell RNA-sequencing data generated in this study were deposited in the NIH Gene Expression Omnibus (GEO) database (https://www.ncbi.nlm.nih.gov/geo) under the accession number GSE276765.

The data discussed in this publication have been deposited in NCBI’s Gene Expression Omnibus and are accessible through GEO Series accession number GSE276775 (https://www.ncbi.nlm.nih.gov/geo/query/acc.cgi?acc=GSE276775) for the bulk RNA-seq experiment and accession number GSE276765 (https://www.ncbi.nlm.nih.gov/geo/query/acc.cgi?acc=GSE276765) for the scRNA-seq experiment.

## SUPPLEMENTAL MATERIAL

10.6084/m9.figshare.26956213 Supplemental Figs. S1–S8: https://doi.org/10.6084/m9.figshare.26956213.

10.6084/m9.figshare.26956237 Supplemental Table S1: https://doi.org/10.6084/m9.figshare.26956237.

## GRANTS

This work was supported by University of Pennsylvania Institute for Infectious and Zoonotic Disease Pilot Grant, University of Pennsylvania, Institute for Translational Medicine and Therapeutics Pilot Grant, NIH NHLBI T32 training grant 5T32-HL007586-38.

## DISCLOSURES

No conflicts of interest, financial or otherwise, are declared by the authors.

## AUTHOR CONTRIBUTIONS

S.K. and A.E.V. conceived and designed research; S.K., M.S., Y.H., A.K., G.Z., J.W., A.I.W., S.A., M.E.G., M.M., N.P.H. and X.L. performed experiments; S.K., M.S., Y.H., A.K., A.E.V., G.Z., J.W., A.I.W., M.E.G., N.P.H. and X.L. analyzed data; S.K. and A.E.V. interpreted results of experiments; S.K. prepared figures; S.K. drafted manuscript; S.K. and A.E.V. edited and revised manuscript; S.K. and A.E.V. approved final version of manuscript.
